# The CHAMP-study: the CHemopreventive effect of lithium in familial AdenoMatous Polyposis; study protocol of a phase II trial

**DOI:** 10.1186/s12876-022-02442-3

**Published:** 2022-08-12

**Authors:** Jasmijn D. G. Linssen, Sanne M. van Neerven, Arthur S. Aelvoet, Clara C. Elbers, Louis Vermeulen, Evelien Dekker

**Affiliations:** 1 Department of Gastroenterology and Hepatology, Amsterdam UMC Location University of Amsterdam, Meibergdreef 9, Amsterdam, The Netherlands; 2 Cancer Center Amsterdam, Laboratory for Experimental Oncology and Radiobiology, Center for Experimental and Molecular Medicine, Amsterdam, The Netherlands; 3 Amsterdam Gastroenterology Endocrinology Metabolism, Laboratory for Experimental Oncology and Radiobiology, Center for Experimental and Molecular Medicine, Amsterdam, The Netherlands; 4 Oncode Institute, Amsterdam, The Netherlands

**Keywords:** Chemoprevention, Colorectal adenomas, Familial adenomatous polyposis, Lithium carbonate

## Abstract

**Background:**

Familial adenomatous polyposis (FAP) is a rare autosomal dominant disease characterized by germline mutations in the *Adenomatous Polyposis Coli* (*APC)* gene, resulting in the development of numerous colorectal adenomas. As these patients have a high risk of developing colorectal cancer (CRC), guidelines suggest prophylactic colectomy during early adulthood, however, adenoma development is still observed in the remaining intestinal tract. Therefore, FAP patients would benefit from chemoprevention strategies reducing the development of adenomas. Recent work in mice reveals a chemopreventive effect of lithium on the development of adenomas by inhibiting the expansion of *Apc* mutated intestinal stem cells (ISCs) within the crypts of normal intestinal mucosa. Here, we aim to investigate the effect of lithium on the spread of *APC* mutant cells within the human intestinal epithelium.

**Methods:**

This prospective phase II single arm trial has a duration of 18 months. FAP patients (18–35 years) with a genetically confirmed *APC* mutation who did not undergo colectomy will be treated with lithium carbonate orally achieving a serum level of 0.2–0.4 mmol/l between month 6 and 12. Colonoscopy with biopsies of normal intestinal mucosa will be performed at baseline and every six months. The primary endpoint is the effect of lithium on the spread of *APC* mutant cells within intestinal crypts over time by using *APC* specific marker *NOTUM *in situ hybridization. Secondary endpoints include change in adenoma burden, patient reported side effects and safety-outcomes. Total sample size is 12 patients and recruitment will take place in the Amsterdam UMC, location AMC in the Netherlands.

**Discussion:**

The outcome of this study will function as a proof-of-concept for the development of novel chemoprevention approaches that interfere with the competition between normal and mutant ISCs.

*Trial registration*: ClinicalTrials.gov (https://clinicaltrials.gov/): NCT05402891 (June 1, 2022) and the EU Clinical Trials Register: EuraCT 2022-000240-30 (January 1, 2022).

**Supplementary Information:**

The online version contains supplementary material available at 10.1186/s12876-022-02442-3.

## Background

Familial adenomatous polyposis (FAP) is an autosomal dominant disorder with a germline mutation in a tumor suppressor gene, *Adenomatous Polyposis Coli* (*APC*) [1]. FAP is a rare condition with an estimated prevalence of 1 out of 5.000–7.000 births [2]. Although FAP is a hereditary disease, it may also be caused by a de novo mutation in the *APC* gene, responsible for approximately 25% of cases [3]. Classical FAP is characterized by the early onset of hundreds to thousands of adenomas in the colon and rectum, and if left untreated, the risk of developing colorectal cancer (CRC) is nearly 100%, occurring between the age of 35 and 45 [2, 4]. To prevent CRC, guidelines suggest prophylactic surgery in these patients dependent on adenoma burden, personal preference and family history [5]. These surgeries are mostly performed around the age of 20, and come with complications and postoperative morbidity [6, 7]. After surgery, new adenomas will develop not only in the remaining parts of the colorectum but in case of pouch creation also in the terminal ileum, in which previously no adenomas were detected, resulting in an ongoing risk of developing intestinal cancer in these patients [8, 9]. Therefore, these patients need lifelong endoscopic surveillance, and potential endoscopic and surgical interventions to prevent intestinal cancer [8, 9]. Patients with FAP would greatly benefit from chemo-preventive strategies that aim to prevent adenoma formation, ultimately resulting in postponing or even avoiding prophylactic surgery. Up to date, several studies on chemo-preventive therapies for patients with FAP were conducted. Due to lack of a significant effect and the adverse events (AE’s) in these patients, no chemo-preventive therapy is routinely used in clinical practice [10–12]. Recent translational work elucidated the molecular mechanism of early oncogenic transformation in FAP and revealed a potential chemopreventive effect of lithium on adenoma development [13, 14]. In the next sections, we will elaborate on this molecular mechanism and potential chemopreventive therapy with lithium.

### *APC*-mutant cells act as supercompetitors

**Fig. 1:**
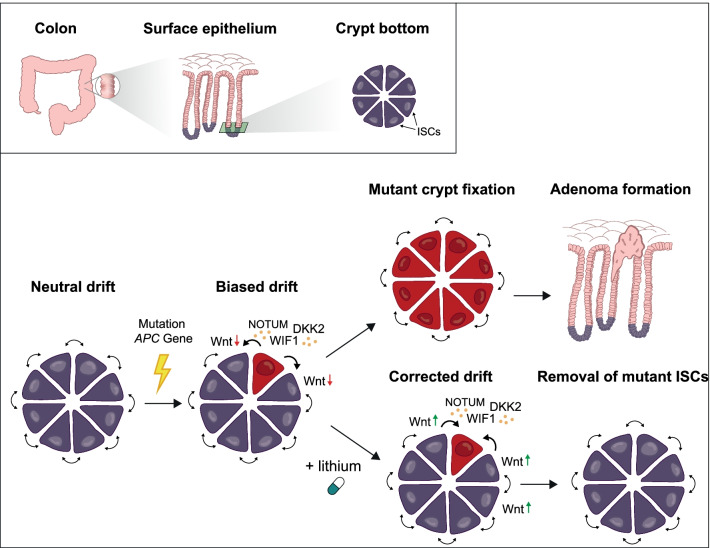
Crypt fixation and chemoprevention with lithium treatment. *APC* = *adenomatous polyposis coli* tumor-suppressor gene. DKK2 = Dickkopf WNT Signaling Pathway Inhibitor 2. ISCs = intestinal stem cells. WIF1 = WNT Inhibitory Factor 1. ISCs reside at the bottom of the crypts of the epithelium of the colon where they engage in ongoing neutral competition with each other for a position in the crypt. These neutral dynamics are disturbed whenever an ISCs acquires a second mutation in the *APC* allele. *APC*-mutant ISCs have a competitive advantage by secreting Wnt antagonists (e.g. NOTUM, WIF1, DKK2) that drive the differentiation of WT ISCs, thereby resulting in mutant crypt fixation and the development of premalignant adenomas. In this study, we propose that boosting the Wnt pathway in WT ISCs using lithium diminishes this competitive advantage and prevents adenoma formation. This figure was created by using Adobe Illustrator 2022 (version 26.0.3)

In FAP, intestinal tumor development is initiated by the loss of the second wild type *APC* allele [15]. The APC protein is a critical negative regulator of the Wnt signaling pathway, an essential signaling route involved in the maintenance of intestinal stem cells (ISCs) which are located in the crypt bottom [16]. Loss of *APC* function results in unrestrained activation of the Wnt signaling cascade which can lead to the development of premalignant adenomas [16]. Previous work demonstrated that ISCs that have acquired an *Apc* mutation have a competitive benefit over wild type (WT) ISCs [15]. As a result, *Apc*-mutant cells gradually replace all WT ISCs, resulting in permanent fixation of a mutation in the crypt bottom and initiation of adenoma formation [13]. Critically, recent studies by us and others have now revealed that *Apc*-mutant ISCs in fact act as supercompetitors, that actively disadvantage WT ISCs [13, 14]. This competitive advantage is due to the expression of Wnt antagonists (e.g. NOTUM, WIF1, DKK2) by *APC*-mutants to which they themselves are insensitive, but result in active differentiation of WT ISCs that are heavily dependent on Wnt signaling (Fig. 1).

### Lithium as chemopreventive therapy

**Fig. 2:**
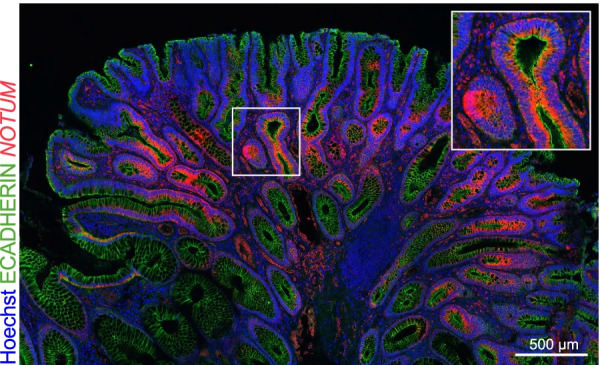
*NOTUM* in situ hybridization (ISH) in human FAP adenomatous lesion. Fluorescent staining of human intestinal adenomas was visualized with a SP8X confocal microscope using Leica Application Suite software (version 3.5.7)

The mechanism by which *APC*-mutant cells outcompete their wild type counterparts immediately suggests a putative target for chemoprevention. Rendering WT ISCs insensitive to the secreted Wnt antagonists by *APC*-mutant cells could prevent further expansion of *APC*-mutant clones. In order to counteract these Wnt antagonists, we proposed to boost the Wnt pathway in WT ISCs using GSK-3β inhibitor lithium, and observed a marked decrease in crypt fixation which consequently resulted in a reduced adenoma burden in a mouse model of FAP [13]. This study implemented a novel way of tracing *Apc-*mutant cells by labeling these cells for Wnt antagonist *Notum*, and analyzing their spread through the crypts over time [13, 14]. In this study in mice, a reduction in the expansion of *Notum* positive clones was observed using a low dose lithium administration (using lithium serum levels of 0.2 mM) [13]. In addition, our work and other studies reveal that *NOTUM* is also highly expressed in crypts and adenomas from FAP patients (Fig. 2) [13, 14, 17]. Until now, no data on lithium use in patients with FAP is available. However, several studies on long term lithium use in patients with bipolar disorders demonstrated a reduced incidence on CRC and cancer in general [18, 19]. Therefore, we propose to trace the spread of *APC*-mutant cells using a similar strategy to assess the effect of low-dose lithium on stem cell competition and number of adenomas in FAP patients.

## Objectives

### Primary objective

The primary objective of this study is to evaluate the effect of low-dose lithium on the spread of *APC*-mutant cells within intestinal crypts of FAP patients over time. This will be assessed by using an *APC* mutation specific marker (*NOTUM*) and is defined as a significant reduction of fixed crypts and reduction of fixed clone size by 50%.

### Secondary objectives

To evaluate:

- The effect of low-dose lithium on number of adenomas and size, determined as sum of diameter of all adenomas;
- Side effects of low-dose lithium in FAP patients, evaluated by lithium side effects questionnaires;
- The safety of low-dose lithium in FAP patients, evaluated by adverse events, findings during physical examination and laboratory tests.

## Methods

### Study design

**Fig. 3:**
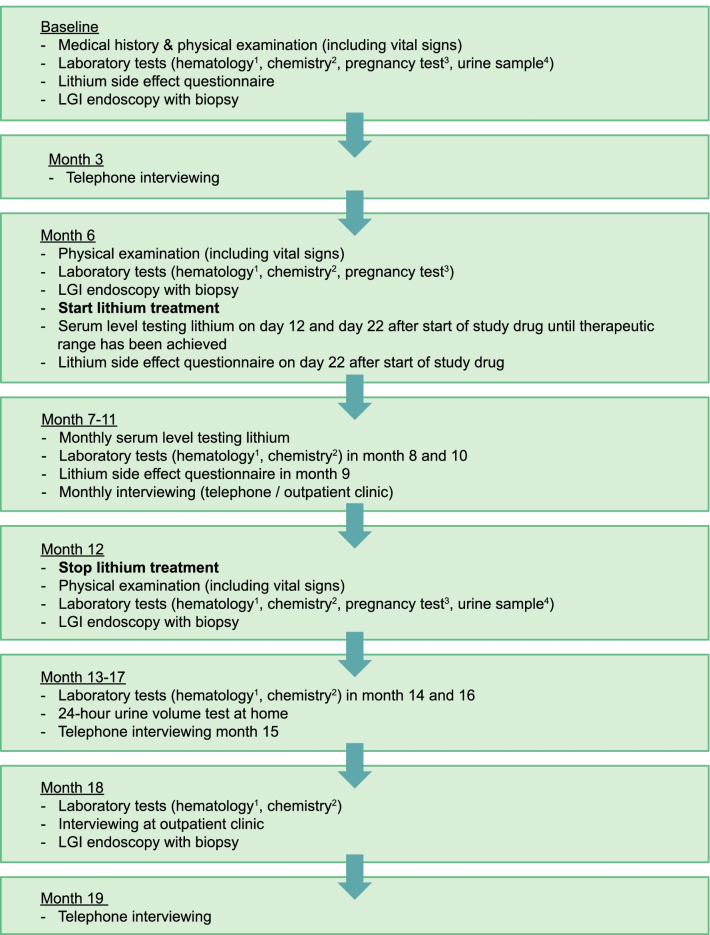
Flowchart of the study design (as of May 2, 2022). ^1^Hematology: haemoglobin, thrombocytes, leukocytes. ^2^Chemistry: GFR, creatinine, urea, sodium, potassium, calcium and TSH (if deviating T4). ^3^A pregnancy test is required for participants with the female gender. ^4^Urine sample: urine osmolality and urine creatinine. This figure was created by using Adobe Illustrator 2022 (version 26.0.3)

The CHAMP-study is a single arm, phase II trial conducted in the Amsterdam UMC, location AMC (AMC) in the Netherlands. The duration of this study will be 18 months in which lithium will be administered between month 6 and 12. The study has been approved by the Medical Ethical Committee of the Amsterdam UMC, location AMC, and has been registered in ClinicalTrials.gov (NCT05402891). This protocol was designed following the Standard Protocol Items: Recommendations for Interventional Trials (SPIRIT). A flowchart of the study design is shown in Fig. 3.

### Study population

Patients diagnosed with FAP, between the age of 18 and 35, with intact colons/rectums, are eligible for this study. No exclusion will be made based on sex or race. For this study no control group will be included, but patients serve as their own control in the non-treatment phase.

### Inclusion criteria

Patients must meet all of the following criteria for inclusion in the study:

- Age between 18 and 35 years;
- Confirmed *APC* germline mutation and one of the following:
  - Minimum of 100 colorectal adenomas;
  - Minimum of 50 colorectal adenomas and a positive family history of a classical FAP phenotype (> 100 colorectal adenomas);
- Intact colon;
- Participant is willing and able to give informed consent for participation.

### Exclusion critera

Patients that meet any of the following criteria will be excluded from participation in this study:

- Participation in any other clinical intervention study; observational trials accepted;
- Lithium use prior to participation of the study;
- Pregnancy, breast-feeding or no use of anticonception;
- No normal intestinal mucosa left for normal tissue biopsy;
- Indication for colectomy within 2 years;
- Known renal impairment, defined as GFR < 60 ml/min;
- Known severe cardiac disorder;
- Known severe brain injury;
- Hypothyroidism;
- Hyponatremia, defined as sodium < 130 mmol/L;
- Positive family history of Brugada syndrome;
- Co-medication known for interacting with lithium;
- Regular non-steroidal anti-inflammatory drug (NSAID) use (defined as more than twice a week for 4 consecutive weeks) within 3 months prior to baseline;
- Use of immunosuppressive or anti-inflammatory drugs within 3 months prior to baseline;
- Use of any other FAP directed drug therapy within 3 months prior to baseline (use of any alternative supplements e.g. turmeric or fish-oil must be noted in patient diary).

### Intervention

Lithium carbonate will be administered by an oral tablet once a day for six months. Subjects will be treated with a starting dose of 200 mg, which will be increased to 300 mg after 5 days. To limit AE’s and side-effects, the lowest effective dose will be administered. The target serum level of lithium is 0.20–0.40 mmol/L and this will be maintained by regular testing of lithium serum levels and dose adjustments if required.

### Outcome measures

#### Primary outcome

The spread of *APC*-mutant cells within intestinal crypts of FAP patients over time will be assessed by quantifying clone sizes as proportions of the crypt circumference positive for *NOTUM* (in parts of eight, 1:8 to 8:8). When a whole crypt is positive for *NOTUM* (8:8), this crypt is fixed (crypt fixation) [12].

#### Secondary outcomes

Secondary endpoints include:

- Difference in number and size of adenomas, determined as sum of diameter of all adenomas between baseline and end of study;
- Patient reported side-effects of low-dose lithium in FAP patients, using a lithium side effect questionnaire (Additional file 1: Lithium side effect questionnaire);
- Safety outcomes of low-dose lithium by analyzing adverse events, physical examination and laboratory findings including hemoglobin, thrombocytes, leukocytes, GFR, creatinine, urea, sodium, potassium, calcium TSH (if deviating T4), urine osmolality and creatinine.

Given the fact that dietary factors can influence intestinal stem cell dynamics, we also aim to monitor the following parameters: number of cups of coffee per day (decaffeinated coffee not included); turmeric use (gram per day); number of fish oil capsules per day (amount of eicosapentaenoic acid in terms of mg) and history of smoking (if yes number of pack years).

### Recruitment, study procedures and assessments

Participants will be recruited from the large cohort of FAP patients at the hereditary GI Cancer clinic at the Amsterdam UMC. Prior to their routine surveillance colonoscopy, patients will be invited to participate by a member of the study team. In total 12 patients will be included in this trial. For all patients, lithium will be administered for a period of six months, between month 6 and 12. Since every participant is given the same treatment, no randomization will take place.

During the course of the study, four colonoscopies (at t = 0 months, t = 6 months, t = 12 months, t = 18 months) will be performed to evaluate the effect of lithium on clone sizes and stem cell dynamics in mucosal biopsies and total adenoma burden. Physical examination (including vital signs) will be performed at baseline and every six months, at time of endoscopy. Laboratory testing will be performed at baseline and every two months from start of lithium treatment. Serum lithium levels will be measured at day 12 and day 22 after start of treatment. When the therapeutic range is reached, serum levels will be measured once a month. Blood samples for determining serum levels must be collected 12 h after oral administration of lithium. If necessary, dose adjustments will be made by the investigator based on serum levels, laboratory tests and side effects. Serum lithium levels will be monitored 12 days after dose adjustments, until the therapeutic range is reached or side-effects diminished. Further check-ups will be performed through telephone interviews to evaluate AE’s and side effects using a lithium side effect questionnaire base on a previously designed tool [20]. A schedule of activities is included in the supplementary materials (Additional file 2: Schedule of activites). More detailed information on study visits, follow-up, data monitoring, safety reporting and auditing is included in the study protocol and clinical trial registry records [21].

#### Assessment of size of fixed clones

For each patient, crypts will be retrieved from biopsies of normal mucosal tissue taken at colonoscopy. *APC*-mutant stem cell dynamics will be determined in these biopsies by tracing the spread of the *APC-*mutant cells using specific expression of *NOTUM* by RNAscope (in situ hybridization). *NOTUM*-positive crypts will be quantified by determining the proportion of the crypt circumference positive for *NOTUM* (in parts of eight, 1:8 to 8:8). This will result in an average clone size distribution for each patient per time point, as well as the proportion of fixed crypts (8:8). By analyzing the differences in clone size distribution before, during and after lithium treatment we aim to observe a relative reduction in average clone size of 50% during the lithium treatment, as well as a reduction in crypt fixation of 50%.

#### Assessment of adenoma burden

A full colonoscopy will be performed at baseline, and at months 6, 12 and 18 to assess adenoma burden and to take biopsies. During colonoscopy, Boston Bowel Preparation Scale (BBPS) should be 2 for each segment and cecal intubation achieved. Adenoma burden will be assessed by estimating the number and size (estimated using a biopsy forceps) of adenomas in each of the 6 colorectal segments: cecum, ascending colon, transverse colon, descending colon, sigmoid and rectum (including retroflexion). For each colonoscopy, the table below will be used to calculate the sum of diameter of adenomas to determine the total colorectal adenoma burden (Table 1). Standard white light endoscopy (WLE) will be used; Narrow band imaging (NBI) is used at the discretion of the endoscopist. Adenomas ≥ 5 mm should be excised at all colonoscopies. At each endoscopy, also two random biopsies of normal mucosa will be taken in each segment, resulting in a total of 12 biopsies of normal intestinal mucosa. Video recordings will be made for all colonoscopies (only using WLE). All four videos of each participant will be reviewed by two separate independent expert endoscopists in a random order, assessing the adenoma burden using the table below. If there is a disagreement of more than 30% in the adenoma burden assessment between the two reviewers, a third independent reviewer will be engaged. Then, the average of these three reviewers will be used for further analysis.

**Table 1:** Total adenoma burden

Segment	Number of adenomas			
	1–2 mm	3–5 mm	6–10 mm	> 10 mm
Cecum				
Ascending colon				
Transverse colon				
Descending colon				
Sigmoid				
Rectum				
Total adenoma burden				

### Data collection and collation

Adenomatous tissue collected endoscopically, as part of routine care, will be stored according to regulations of the department of pathology in the AMC. Normal intestinal mucosa collected endoscopically, as part of study material to assess *NOTUM* distribution size e.g., will be analysed in the Center for Experimental and Molecular Medicine (CEMM). Source documents and CRFs will be stored by the project leader for 15 years after closure of the trial. Collected samples will be stored for 5 years. In case of informed consent is reached for participation in a biobank, samples will be stored for 15 years. Data of the subjects will be coded in order of participation. The code and the data are stored in different locations. The code can only be seen by the investigators. Qualified authorities can get insight in the code and data, but only when accompanied by the investigators. Informed consent forms are kept in separate files, to ensure the data security. The handling of personal data will comply with the Dutch Personal Data Protection Act (in Dutch: De Wet Algemene Verordening Gegevensbescherming, AVG).

### Data analysis

All *NOTUM* + clone size analysis data and adenoma counts will be scored blindly. To describe the study population, baseline characteristics and changes in outcome parameters during follow-up, descriptive statistics will be used in this study. SPSS for Windows software (Chicago, IL, USA) version 26.0 will be used for these analyses. If other statistical analysis will be used, this will be mentioned in the study paper. Primary analysis for the primary outcome parameter will be done by analyzing the data using two-sided student’s t-test. The limit for the statistical significance will be established at *p* < 0.05 with a confidence interval of 95%. Descriptive statistics will be used to describe the changes in secondary outcome measures during follow-up.

### Sample size calculation

Simulations of clonal expansion in the intestine indicate that in order to detect a reduction of 50% (power stat. 80%, *p* = 0.05) in fitness of *APC*-mutant cells, we need to analyze sizes of *APC* mutant clones within 11 partially populated crypts at 4 time points in 12 individuals. To ensure sufficient material we need 840 crypts per patient per time point. A typical biopsy yields 100–250 crypts, therefore we will perform 12 biopsies in total (2 biopsies per segment, 6 segments) per patient per time point. In that way, sufficient data will be obtained to confirm or refute the study hypothesis.

## Discussion

Patients with FAP develop numerous adenomatous lesions in the colon and rectum leading to a high risk of CRC. To reduce this risk, guidelines recommend endoscopic surveillance and prophylactic surgery which is usually performed at a relatively young age [5]. Surgery is invasive and comes with the risk of complications and reduced functional outcome [6, 7]. Moreover, adenoma formation will continue in the remaining proximal and distal intestinal tract requiring lifelong endoscopic surveillance and potentially further endoscopic or surgical treatments [8, 9]. Therefore, patients with FAP would benefit from pharmacological therapy with a chemopreventive effect on the development of adenomas. Controlling the development of adenomas could potentially lead to extending endoscopic surveillance intervals, reducing endoscopic interventions and postponing or avoiding surgical interventions. If lithium treatment results in a reduced *NOTUM* expansion and the drug is safe with few side-effects, a larger-scale clinical trial could be initiated.

To prevent adenoma formation, the timing of pharmacological intervention is crucial since our proposed chemoprevention strategy is designed to interfere with ISC dynamics before crypt fixation has taken place. As such, a study on lithium in *Apc*^*min*^ mice showed no significant effect of lithium on adenoma numbers, potentially because therapy was started after *Apc*-deficient clones reached fixation within crypts [22]. Importantly, no increase in adenoma size or numbers was seen either [22]. This supports the safety of the current study and the notion that chemoprevention should be initiated at an early age [13].

Several other chemoprevention-studies in FAP patients have been reported. In a randomized, double-blind, phase III trial in 171 patients, no significant effect was seen when were treated with the combination of eflornithine and sulindac compared to the treatment with each drug separately [23]. A randomized controlled trial on the effect of celexocib in 77 FAP patients reported a significant reduction in number of adenomas in the colorectum after treatment for six months, leading to the use of celexocib in the clinic in FAP patients. Eventually, the FDA withdrew its approval due to the lack of sufficient evidence on the efficacy and safety of celexocib in the clinic and the increased risk for cardiovascular and gastrointestinal side effects [24, 25]. A study evaluating the effect of sulindac and erlotinib compared to placebo in 92 patients observed a decrease in adenoma burden in the colorectum. However, the (preventive) effect on adenoma formation is undefined. Subsequently, side-effects and AEs were reported at a significant level and there is no data on long-term effect [26]. In a small pilot study on the effect of sirolimus in four FAP patients, a decrease in number of adenomas was seen. However, these results did not outweigh the numerous reported AEs, causing 1 of 4 participants to terminate the medication prematurely [11].

In this type of studies, accurate, non-biased and observer-independent assessment of this primary endpoint is challenging. In the previously mentioned clinical studies, the primary endpoints were number and/or size of adenomas as determined by photographs or video recordings (whether or not marked by tattoo dye). Despite that these recordings were blindly reviewed by multiple endoscopic experts, it remains challenging to quantify adenoma burden of up to a thousand adenomas in the entire colorectum based on video recordings. For our study, we decided to assess the effect of lithium by NOTUM-expression within crypts. This seems a more objective method and will also provide relevant translational data. Additionally, the effect on adenoma development and burden will be assessed by endoscopists who will review video recordings of the colonoscopies in a random order. The combination of the primary study parameter and the secondary study parameters, obtained through endoscopic measurements and laboratory findings, will provide both clinical and translational information.

In prior studies, numerous reported AEs and side-effects did not outweigh the clinical benefit of the drug in FAP patients leading to a high dropout rate and a limit use of these drugs in the clinic [11, 24, 26]. These reported AEs were mainly due the administration of relative high dosage of the drugs. In this study lithium will be investigated, which is used for several decades and extensive studies have been conducted on its pharmacodynamics and pharmacokinetics. In this trial lithium carbonate will be administered orally in a dosage of 300 mg a day for a short period of time (6 months), which correlates with a serum level of 0.2–0.4 mmol/L. The incidence of side-effects and AEs in patients treated with lithium is associated with a much higher serum level and long-term use [20, 27, 28]. Serum levels of participants in this study will be regularly monitored. Further safety monitoring will be achieved through telephonic interviewing, physical examination and laboratory testing. By doing so, side-effects and toxicity can be minimalized or caught at an early stage leading to a relative low number of reported AE’s.

Taken together, this study provides a proof of concept for the development of chemoprevention strategies that focus on modulating the competition between WT and mutant ISCs and, in this particular example, could potentially lead to the development of Wnt antagonist inhibitors in the future. Prevention of adenoma development in FAP patients could lead to extending endoscopic surveillance intervals and postponing or avoiding surgical intervention. Importantly, this strategy might also be useful to prevent expansion of premalignant clones in other hereditary cancer syndromes.

## Supplementary Information

**Additional file 1.** Lithium side effect questionnaire

**Additional file 2.** Schedule of activities

## Data Availability

After completion and publication of the trial coded data will be available from the corresponding author on reasonable request.

## Abbreviations

AE: Adverse event
*APC*: *Adenomatous polyposis coli* Tumor-suppressor gene
BBPS: Boston Bowel Preparation Scale
CEMM: Center for Experimental and Molecular Medicine
CRC: Colorectal cancer
DKK2: Dickkopf WNT Signaling Pathway Inhibitor 2
FAP: Familial adenomatous polyposis
FDA: Food and drug administration
ISC: Intestinal stem cell
ISH: In situ hybridization
NBI: Narrow band imaging
NSAID: Non-steroidal anti-inflammatory drug
SPSS: Statistical Package for the Social Sciences
WIF1: WNT Inhibitory Factor 1
WLE: White light endoscopy
WT: Wild type

## References

[1] Kinzler KW, Nilbert MC, Su LK, Vogelstein B, Bryan TM, Levy DB (1991). Identification of FAP locus genes from chromosome 5q21. Science.

[2] Bussey HJ, Veale AM, Morson BC (1978). Genetics of gastrointestinal polyposis. Gastroenterology.

[3] Bisgaard ML, Fenger K, Bülow S, Niebuhr E, Mohr J (1994). Familial adenomatous polyposis (FAP): frequency, penetrance, and mutation rate. Hum Mutat.

[4] Bussey HJ (1975). Familial polyposis coli: family studies, histopathology, differential diagnosis and results of treatment.

[5] van Leerdam ME, Roos VH, van Hooft JE, Dekker E, Jover R, Kaminski MF (2019). Endoscopic management of polyposis syndromes: European Society of Gastrointestinal Endoscopy (ESGE) guideline. Endoscopy.

[6] Pasquer A, Benech N, Pioche M, Breton A, Rivory J, Vinet O (2021). Prophylactic colectomy and rectal preservation in FAP: systematic endoscopic follow-up and adenoma destruction changes natural history of polyposis. Endosc Int Open.

[7] Kartheuser A, Stangherlin P, Brandt D, Remue C, Sempoux C (2006). Restorative proctocolectomy and ileal pouch-anal anastomosis for familial adenomatous polyposis revisited. Fam Cancer.

[8] Tajika M, Tanaka T, Ishihara M, Hirayama Y, Oonishi S, Mizuno N (2019). Long-term outcomes of metachronous neoplasms in the ileal pouch and rectum after surgical treatment in patients with familial adenomatous polyposis. Endosc Int Open.

[9] Friederich P, de Jong AE, Mathus-Vliegen LM, Dekker E, Krieken HH, Dees J (2008). Risk of developing adenomas and carcinomas in the ileal pouch in patients with familial adenomatous polyposis. Clin Gastroenterol Hepatol.

[10] Ricciardiello L, Ahnen DJ, Lynch PM (2016). Chemoprevention of hereditary colon cancers: time for new strategies. Nat Rev Gastroenterol Hepatol.

[11] Roos VH, Meijer BJ, Kallenberg FGJ, Bastiaansen BAJ, Koens L, Bemelman FJ (2020). Sirolimus for the treatment of polyposis of the rectal remnant and ileal pouch in four patients with familial adenomatous polyposis: a pilot study. BMJ Open Gastroenterol.

[12] Hull MA, Sprange K, Hepburn T, Tan W, Shafayat A, Rees CJ (2018). Eicosapentaenoic acid and aspirin, alone and in combination, for the prevention of colorectal adenomas (seAFOod Polyp Prevention trial): a multicentre, randomised, double-blind, placebo-controlled, 2 × 2 factorial trial. Lancet.

[13] van Neerven SM, de Groot NE, Nijman LE, Scicluna BP, van Driel MS, Lecca MC (2021). Apc-mutant cells act as supercompetitors in intestinal tumour initiation. Nature.

[14] Flanagan DJ, Pentinmikko N, Luopajärvi K, Willis NJ, Gilroy K, Raven AP (2021). NOTUM from Apc-mutant cells biases clonal competition to initiate cancer. Nature.

[15] Vermeulen L, Morrissey E, van der Heijden M, Nicholson AM, Sottoriva A, Buczacki S (2013). Defining stem cell dynamics in models of intestinal tumor initiation. Science.

[16] Schneikert J, Behrens J (2007). The canonical Wnt signalling pathway and its APC partner in colon cancer development. Gut.

[17] Kleeman SO, Koelzer VH, Jones HJ, Vazquez EG, Davis H, East JE (2020). Exploiting differential Wnt target gene expression to generate a molecular biomarker for colorectal cancer stratification. Gut.

[18] Martinsson L, Westman J, Hällgren J, Ösby U, Backlund L (2016). Lithium treatment and cancer incidence in bipolar disorder. Bipolar Disord.

[19] Huang RY, Hsieh KP, Huang WW, Yang YH (2016). Use of lithium and cancer risk in patients with bipolar disorder: population-based cohort study. Br J Psychiatry.

[20] Wilting I, Heerdink ER, Mersch PP, den Boer JA, Egberts AC, Nolen WA (2009). Association between lithium serum level, mood state, and patient-reported adverse drug reactions during long-term lithium treatment: a naturalistic follow-up study. Bipolar Disord.

[21] ClinicalTrials.gov: The CHAMP-study: The CHemopreventive Effect of Lithium in Familial AdenoMatous Polyposis (Lithium in FAP) [Internet]. 2022. https://clinicaltrials.gov/ct2/show/study/NCT05402891.

[22] Gould TD, Gray NA, Manji HK (2003). Effects of a glycogen synthase kinase-3 inhibitor, lithium, in adenomatous polyposis coli mutant mice. Pharmacol Res.

[23] Burke CA, Dekker E, Lynch P, Samadder NJ, Balaguer F, Hüneburg R (2020). Eflornithine plus sulindac for prevention of progression in familial adenomatous polyposis. N Engl J Med.

[24] Steinbach G, Lynch PM, Phillips RK, Wallace MH, Hawk E, Gordon GB (2000). The effect of celecoxib, a cyclooxygenase-2 inhibitor, in familial adenomatous polyposis. N Engl J Med.

[25] European Medicines Agency concludes on use of celecoxib in familial adenomatous polyposis: Celecoxib not to be used off-label following Onsenal withdrawal [Internet]. [Press release]. London: European Medicines Agency, 2011 May 20.

[26] Samadder NJ, Kuwada SK, Boucher KM, Byrne K, Kanth P, Samowitz W (2018). Association of sulindac and erlotinib vs placebo with colorectal neoplasia in familial adenomatous polyposis: secondary analysis of a randomized clinical trial. JAMA Oncol.

[27] Azab AN, Shnaider A, Osher Y, Wang D, Bersudsky Y, Belmaker RH (2015). Lithium nephrotoxicity. Int J Bipolar Disord.

[28] Tondo L, Abramowicz M, Alda M, Bauer M, Bocchetta A, Bolzani L (2017). Long-term lithium treatment in bipolar disorder: effects on glomerular filtration rate and other metabolic parameters. Int J Bipolar Disord.

