# Supplementary material for: The CHAMP-study: the CHemopreventive effect of lithium in familial AdenoMatous Polyposis; study protocol of a phase II trial
Source: BMC Gastroenterol. 2022 Aug 12;22:383. doi: 10.1186/s12876-022-02442-3 (PMC9373414; doi:10.1186/s12876-022-02442-3)
Supplement: Supplementary file 1 — Additional file 1. Lithium side effect questionnaire [file 12876_2022_2442_MOESM1_ESM.docx]

**Additional file 1: Lithium side effect questionnaire**

Please indicate to what extent you suffer from the mentioned symptoms, it is only about your own experience.

|  | No | Light, hardly any disturbance | Moderate, some disturbance | Heavily with much disturbance |
| --- | --- | --- | --- | --- |
| Headache | 0 | 1 | 2 | 3 |
| Dizziness | 0 | 1 | 2 | 3 |
| Fatigue | 0 | 1 | 2 | 3 |
| Blurred double vision | 0 | 1 | 2 | 3 |
| Concentration problems | 0 | 1 | 2 | 3 |
| Memory disorders | 0 | 1 | 2 | 3 |
| Nausea | 0 | 1 | 2 | 3 |
| Dry mouth | 0 | 1 | 2 | 3 |
| Thirsty | 0 | 1 | 2 | 3 |
| Increased urination | 0 | 1 | 2 | 3 |
| Reduced appetite | 0 | 1 | 2 | 3 |
| Increased appetite | 0 | 1 | 2 | 3 |
| Diarrhea | 0 | 1 | 2 | 3 |
| Obstipation | 0 | 1 | 2 | 3 |
| Sexual dysfunction | 0 | 1 | 2 | 3 |
| Sweating | 0 | 1 | 2 | 3 |
| Tremor of the hands | 0 | 1 | 2 | 3 |
| Muscle weakness | 0 | 1 | 2 | 3 |
| Coordination disorder | 0 | 1 | 2 | 3 |
