# Supplementary material for: The CHAMP-study: the CHemopreventive effect of lithium in familial AdenoMatous Polyposis; study protocol of a phase II trial
Source: BMC Gastroenterol. 2022 Aug 12;22:383. doi: 10.1186/s12876-022-02442-3 (PMC9373414; doi:10.1186/s12876-022-02442-3)
Supplement: Supplementary file 2 — Additional file 2. Schedule of activities [file 12876_2022_2442_MOESM2_ESM.docx]

**Additional file 2: Schedule of Activities**

| **Visit** | **Screening (<30 days prior to day 1)** | **Trial month^a^** | | | | | | | | | | | | | | | **Follow-up (30 days after last colonoscopy)** |
| --- | --- | --- | --- | --- | --- | --- | --- | --- | --- | --- | --- | --- | --- | --- | --- | --- | --- |
| **Study procedures** |  | **0** | **3** | **6 SoT** | **6 (Day 12)** | **6 (Day 22)** | **7** | **8** | **9** | **10** | **11** | **12 EoT** | **14** | **15** | **16** | **18** |  |
| **Site Visit** |  | X |  | X |  |  |  | X |  | X |  | X |  |  |  | X |  |
| **Administrative** | | | | | | | | | | | | | | | | | |
| Informed consent | X | X |  |  |  |  |  |  |  |  |  |  |  |  |  |  |  |
| Inclusion/exclusion criteria | X | X |  |  |  |  |  |  |  |  |  |  |  |  |  |  |  |
| Medical history and demographics | X | X |  |  |  |  |  |  |  |  |  |  |  |  |  |  |  |
| **Safety Assessments** | | | | | | | | | | | | | | | | | |
| Physical exam, weight, vital signs |  | X |  | X |  |  |  |  |  |  |  | X |  |  |  |  |  |
| Lithium side-effect questionnaire |  | X |  |  |  | X |  |  | X |  |  |  |  |  |  |  |  |
| Telephone interviewing |  |  | X |  |  |  | X |  | X |  | X |  |  | X |  |  | X |
| **Efficacy Assessments** | | | | | | | | | | | | | | | | | |
| Colonoscopy with biopsies |  | X |  | X |  |  |  |  |  |  |  | X |  |  |  | X |  |
| **Laboratory Assessments** | | | | | | | | | | | | | | | | | |
| Hematology, Renal function, Elektrolytes, (Para)thyroid function) ^b^ |  | X |  | X^d^ |  |  |  | X |  | X |  | X | X |  | X | X |  |
| Pregnancy test ^c^ |  | X |  | X^d^ |  |  |  |  |  |  |  | X |  |  |  |  |  |
| Spot urine sample |  | X |  |  |  |  |  |  |  |  |  | X |  |  |  |  |  |
| 24-hour urine volume test at home |  |  |  |  |  |  |  |  |  |  |  |  |  | X |  |  |  |
| **Study Drug Administration** | | | | | | | | | | | | | | | | | |
| Dispense study drug |  |  |  | X |  |  |  | X |  | X |  |  |  |  |  |  |  |
| **Pharmacokinetics^e^** | | | | | | | | | | | | | | | | | |
| Serum level Lithium |  |  |  |  | X | X | X | X | X | X | X | X |  |  |  |  |  |
| SoT= Start of Treatment; EoT =End of Treatment  ^a^ Visits should occur within ± 7 days of scheduled date. Hematology: haemoglobin, thrombocytes, leukocytes  ^b^ Chemistry: GFR, creatinine, urea, sodium, potassium, calcium and TSH (if deviating T4)  ^c^A pregnancy test for women is required at screening, prior to start of the study drug (at site) and at the end of the treatment. within 72 hours prior to the first dose of the study drug.  ^d^ Hematology, chemistry and pregnancy test samples in months 6 must be collected prior to first administration of study drug.  ^e^ Pharmacokinetic samples should always be collected postdose, with a minimum of 12 hours.  All reasonable attempts should be made to collect samples at scheduled time points and record actual times. | | | | | | | | | | | | | | | | | |
